# Highly efficient DNA-free gene disruption in the agricultural pest *Ceratitis capitata* by CRISPR-Cas9 ribonucleoprotein complexes

**DOI:** 10.1038/s41598-017-10347-5

**Published:** 2017-08-30

**Authors:** Angela Meccariello, Simona Maria Monti, Alessandra Romanelli, Rita Colonna, Pasquale Primo, Maria Grazia Inghilterra, Giuseppe Del Corsano, Antonio Ramaglia, Giovanni Iazzetti, Antonia Chiarore, Francesco Patti, Svenia D. Heinze, Marco Salvemini, Helen Lindsay, Elena Chiavacci, Alexa Burger, Mark D. Robinson, Christian Mosimann, Daniel Bopp, Giuseppe Saccone

**Affiliations:** 1 Department of Biology, University of Naples “Federico II”, 80126 Napoli, Italy; 2 Institute of Biostructures and Bioimaging (IBB), CNR, 80134 Naples, Italy; 3 Department of Pharmacy, University of Naples “Federico II”, 80134 Napoli, Italy; 4 Department of Physics “E. Pancini”, University of Naples “Federico II”, 80126 Napoli, Italy; 5 Stazione Zoologica Anton Dohrn, Center Villa Dohrn for Benthic Ecology, Punta San Pietro, 80077 Ischia, Italy; 6 Institute of Molecular Life Sciences, University of Zürich, Zürich, 8057 Switzerland; 7 SIB Swiss Institute of Bioinformatics, University of Zürich, Zürich, 8057 Switzerland

## Abstract

The Mediterranean fruitfly *Ceratitis capitata* (medfly) is an invasive agricultural pest of high economic impact and has become an emerging model for developing new genetic control strategies as an alternative to insecticides. Here, we report the successful adaptation of CRISPR-Cas9-based gene disruption in the medfly by injecting *in vitro* pre-assembled, solubilized Cas9 ribonucleoprotein complexes (RNPs) loaded with gene-specific single guide RNAs (sgRNA) into early embryos. When targeting the eye pigmentation gene *white eye* (*we*), a high rate of somatic mosaicism in surviving G0 adults was observed. Germline transmission rate of mutated *we* alleles by G0 animals was on average above 52%, with individual cases achieving nearly 100%. We further recovered large deletions in the *we* gene when two sites were simultaneously targeted by two sgRNAs. CRISPR-Cas9 targeting of the *Ceratitis* ortholog of the *Drosophila* segmentation *paired* gene (*Ccprd*) caused segmental malformations in late embryos and in hatched larvae. Mutant phenotypes correlate with repair by non-homologous end-joining (NHEJ) lesions in the two targeted genes. This simple and highly effective Cas9 RNP-based gene editing to introduce mutations in *C. capitata* will significantly advance the design and development of new effective strategies for pest control management.

## Introduction

The Mediterranean fruitfly *Ceratitis capitata* (medfly) is an economically relevant agricultural pest infesting more than 260 crop species including fruits, vegetables, and nuts^1^. Wild populations can be contained by the Sterile Insect Technique (SIT), an eradication strategy based on the repeated release of large numbers of factory-grown sterile males into infested areas^2, 3^. *C. capitata* was the first non-Drosophilidae insect species in which transposon-mediated germline transformation was established^4, 5^. Various transgenic strains have been developed to improve SIT and other pest control strategies^6–14^. Furthermore, embryonic RNA interference was successfully applied to study *in vivo* functions of key *Ceratitis* genes controlling sex determination^15, 16^.

Nonetheless, a more comprehensive study of gene functions in *Ceratitis* will be needed to further improve existing control strategies. To generate long-lasting and heritable changes in gene function, the novel CRISPR-Cas9 gene editing system with its modular and simple components provides a promising tool for reverse genetics also in insects and to implement scalable and reproducible pest control strategies^17, 18^. In short, Cas9 endonuclease recognizes a specific genomic region based on sequence complementary of a preassembled chimeric single guide RNA (sgRNA), and induces double-strand DNA breaks (DSBs) at the targeted site. DSBs activate non-homologous end-joining (NHEJ) or homology-directed (HR) DNA repair, two cellular events which can be exploited not only to disrupt genes but also to modify sequences after a given DNA template is provided or to introduce exogenous sequences^19, 20^.

In the model insect *Drosophila melanogaster*, the successful use of the Cas9 system to introduce genome modifications was based on injecting different combinations of CRISPR-Cas9 reagents into embryos, such as (1) DNA plasmids expressing Cas9 protein and sgRNA^21^, (2) DNA plasmid transcribing sgRNA (or *in vitro* synthesized sgRNA) into transgenic flies that express Cas9 in the embryonic germ line^22–24^ and (3) *in vitro*-transcribed Cas9 mRNA and sgRNA^25, 26^. Furthermore, transgenic *Drosophila* strains have been generated that express both Cas9 and sgRNA and can induce a variety of genetic effects beyond the mere gene editing^27^.

The efficiencies of these different application modes have been tested over the past few years. For instance, injections of purified Cas9 protein preloaded with trRNA, trans-activating RNA and gene-specific crRNA into *Drosophila* embryos yielded biallelic mutations in more than 8% and germ line transmission in more than 17% of the injected individuals^28^. The model lepidopteran *Bombyx mori* was the first non Drosophilidae insect species in which CRISPR-Cas9, applied co-injecting mRNA/sgRNAs, was shown to be highly effective also in inducing large deletions (95% somatic G0 mutants and 36% germ line transmission rate)^29^. In the mosquito *Aedes aegypti*, injections of preassembled RNPs with commercially purified Cas9 produced detectable lesions in 50% of injected individuals and germline transmission rate was 90%^30^. Furthermore, this study demonstrated that sgRNAs were more effective than the dual component system with crRNA and trRNA.

Altogether the use of CRISPR-Cas9 for gene editing was shown to be effective in more than two dozen arthropod species, belonging to 6 different orders (Diptera, Lepidotera, Coleoptera, Orthoptera, Decapoda and Diplostraca)^18, 31, 32^. In most of these studies, the preferred approach was to deliver mRNA encoding Cas9 together with synthetic gRNAs (or produced by a U6-Promoter DNA plasmid). A drawback of this method is that, because of the extended time needed for Cas9 translation, folding and interaction with gRNAs, only dividing cells that accumulate sufficient levels RNPs can be efficiently targeted. Injecting commercially available Cas9 protein instead of mRNA yielded higher rates of NHEJ mediated lesions in *Aedes aegypti* ^30, 33^, in three Anopheles species (*Anopheles albimanus*, *Anopheles coluzzii*, and *Anopheles funestus*)^34^ and in three lepidopteran species (*Vanessa carduii*, *Junonia coenia* and *Papilio xuthus*)^35, 36^. Lab-purified recombinant Cas9 was also very effective in generating mutants in the crop pest moth *Spodoptera littoralis*, resulting in mono/biallelic mutations in 90% of the injected G0 generation and germline transmission rate was 43%^37^.

The objectives of our study were to provide a protocol for production and purification of recombinant Cas9 protein and to demonstrate that injecting RNPs results in highly efficient gene disruption in the major agricultural pest *C. capitata*. We decided on the strategy of injecting Cas9 RNPs into *Ceratitis* embryos for the following reasons: (1) lab-produced Cas9 protein is cheaper than commercially available protein and also more cost-effective than the use of *in vitro* synthetized Cas9 mRNA^28, 37, 38^; (2) preloaded Cas9 RNPs can act immediately following injection^33, 34^ and this often results in higher efficiencies of inducing biallelic mutations in both somatic and germline cells^28, 33, 34, 38^; (3) there are potentially less off-target events^37, 39, 40^; (4) Cas9 protein is more stable and robust than synthetic Cas9 mRNA, and (5) a genetic strategy free of exogenous DNA in pest control management will be more acceptable avoiding contentious regulatory issues^18, 31^.

To test the feasibility of Cas9-mediated gene disruption in the medfly, we targeted the *Ceratitis* orthologues of *white* and *paired* genes, which in *D. melanogaster* are required for eye pigmentation^4, 5, 41^ and for embryonic segmentation^42, 43^, respectively.

The *we* gene is an ideal target to test Cas9-mediated disruption for the following reasons: (1) *we* function is cell-autonomous and unpigmented mutant cells can be readily detected in the adult eye; (2) a *we* mutant *Ceratitis* strain (*w* ^1^ */w* ^1^)^41^ is available to test new loss-of-function alleles for complementation. The zygotic activity of the *Drosophila paired* gene (*prd*) is required for proper segmentation of the developing embryo^42^. *Drosophila* embryos homozygous for *prd* loss-of-function alleles lack every other segment and die before hatching (pair-rule phenotype)^42^. Early segmentation is a well-conserved process in higher dipterans^43, 44^; we therefore hypothesized that somatic lesions in the *prd* ortholog (*Ccprd*; Genbank JAB89073.1) will result in comparable segmental defects and early larval lethality in *Ceratitis*.

Here, we show that lab-purified recombinant Cas9 protein *in vitro* pre-assembled with sgRNA is highly effective at inducing mono- and bi-allelic lesions in both somatic and germline cells. Our results also confirm a conserved function of the *Ccprd* in proper embryonic segmentation.

## Results

### Cas9-induced somatic disruption of the *we* gene

**Figure 1:**
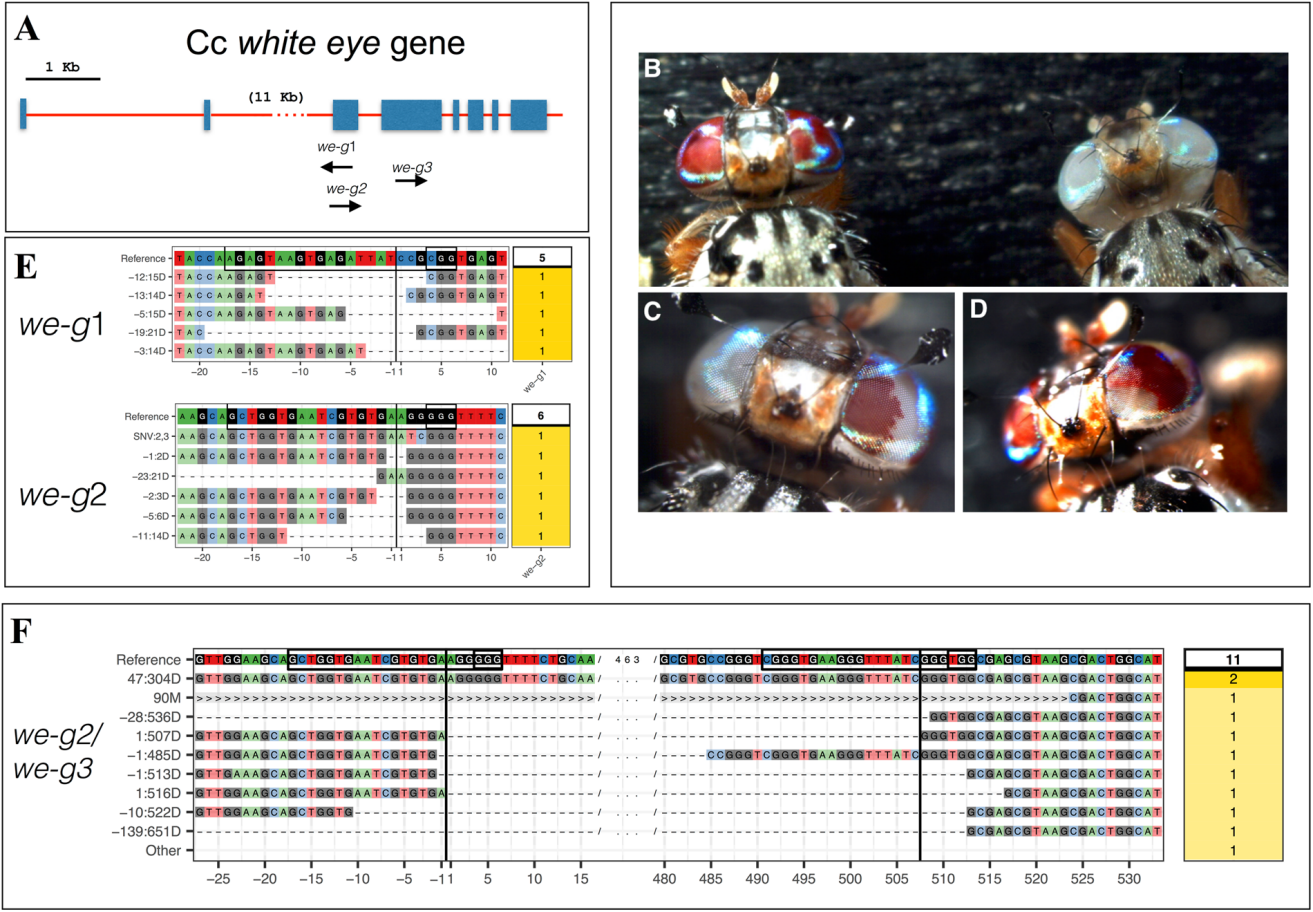
CRISPR-Cas9 targeting of *we* gene. (**A**) A scheme of the genomic organization of the *we* gene and the 3 sgRNAs targeted sites (denoted *we-g*1, *we-g2*, *we-g3*) (**B**) wildtype *we* ^+^ */we* ^+^ (left) and mutant *we* (*w* ^1^ */w* ^1^)^41^ individual (right). (**C**) and (**D**) examples of somatic *we* clones in the eyes of G0 individuals. (**E**) Sequences of mutant *we* alleles recovered from G0 individuals targeted with *we-g*1 or *we-g*2. The CrispRVariants plots^67^ show sequence composition of various alleles compared to reference (target sequence and PAM are boxed and position of cut site is indicated with a black line). The label of individual allele denotes the location (relative to the cut site) and the size of the deletion. Number of sequenced clones per allele are shown in the yellow box. (**F**) Sequences of mutant *we* alleles in G0 individuals targeted with duplex *we-g*2 and *we-g*3 RNPs. The CrispRVariants plot shows the spectrum of induced deletions in G0 individuals. Black lines indicate the position of the cut site in each target sequence (black boxes). >symbol signifies absence of mapped sequence from a partial alignment.

We produced our own supply of Cas9 endonuclease by expressing and purifying HIS tagged recombinant protein from *Escherichia coli* ^28^, following a customary purification protocol which is described in detail in the material and method section^45, 46^. We used the CHOPCHOP software^47^ to identify potential Cas9 target sequences in *we* and to design three independent sgRNAs, *we*-g1, *we*-g2, and *we*-g3 (Fig. 1A). Injections with unloaded recombinant Cas9 protein caused an almost twofold lower survival rate (15–18%, respectively with 0.9 and 3.6 μg/μl Cas9) compared to buffer alone injections (30%), suggesting a measurable level of toxicity of Cas9 protein in *Ceratitis* embryos (Supplementary Table 1). In contrast, an intermediate Cas9 concentration showed higher survival rate similar to the one with only buffer (28%). We cannot exclude that also differences in the technical execution of the embryos microinjections added further variability. A slightly more pronounced toxicity of purified Cas9 was observed during transition from larvae to pupae (survival rates: 37–49% compared to 84%).

We next loaded recombinant Cas9 protein with individual sgRNAs *in vitro* (Cas9 1.8 μg/μl and gRNAs 0.2 μg/μl) and injected the RNP complexes into early syncytial embryos of the wildtype Benakeion strain. We aimed at targeting syncytial nuclei to maximize the efficiency of inducing NHEJ lesions. A biallelic hit (*we* is autosomal in *Ceratitis*) at this early stage is expected to produce large clones of mutant tissue in injected individuals^28, 34, 37–39^. In a first experiment, we injected *we*-g1 RNPs in a buffer containing 300 mM KCl to improve solubilization of Cas9, as previously established to achieve maximal Cas9 activity in zebrafish^39^. Of 240 injected embryos, 134 larvae hatched and only 6 survived to adulthood (Supplementary Table 2). Such a low survival rate was due to technical problems related to this specific set of injections and larvae rearing. Three out of six (50%) displayed a mosaic pattern of *white* unpigmented ommatidia surrounded by wild-type pigmented ones (Fig. 1B–D). One of the two eyes in one individual was completely white, suggesting that a biallelic gene disruption event occurred at an early stage in the primordial lineage (Fig. 1C). The presence of mutations was confirmed by sequencing of PCR products spanning the cleavage sites. Direct sequencing of amplified genomic DNA showed heterogeneity in nucleotide calls around the cleavage site close to the protospacer-adjacent motif (PAM) site, consistent with a range of different NHEJ-induced alterations. Genomic *we* PCR products were obtained from pools of injected larvae or single adult G0 flies. Indels, mostly deletions, of variable length (Fig. 1E; *we*-g1, 14–21 bp), were detected in cloned fragments, consistent with previous studies^17, 18^. Embryos injections with *we-*g2 (150 mM KCl), and *we-*g3 (300 mM KCl) led to 13 and 10% adult survival rates, respectively, and to a lower percentage of adults with eye pigmentation mosaicism (4% for *we*-g2) or none (*we*-g3) (Supplementary Table 2).

Injections of *we*-g1 + *we*-g2 RNPs (150 mM KCl) led to 23% adult mosaics (Supplementary Table 2), but this duplex RNP targeting did not produce deletions between the two targeted sites which are 96 bp apart; furthermore, only *we*-g2 induced indels were observed (reported in Fig. 1E) (Supplementary Table 2). Again, sequencing of cloned PCR products revealed NHEJ deletions, ranging from 2–21 bp, in proximity to the PAM site of *we*-g2. Three additional rounds of injections of *we*-g1/*we*-g2 into a total of 600 embryos (data not shown), raising the buffer to 300 mM KCl, confirmed the lack of deletions between the two targeted sites by PCR analysis on genomic DNA from pools of larvae. When injecting simultaneously *we*-g2 and *we*-g3 RNPs (in 300 mM KCl) in early embryos, we did recover deletions spanning the 2 more distant targeted sites, which are 436 bp apart. 9 deletions events ranging from 304 bp to 651 bp were observed by cloning and sequencing (Fig. 1F).

### Cas9-induced germ line disruption of the *we* gene

**Figure 2:**
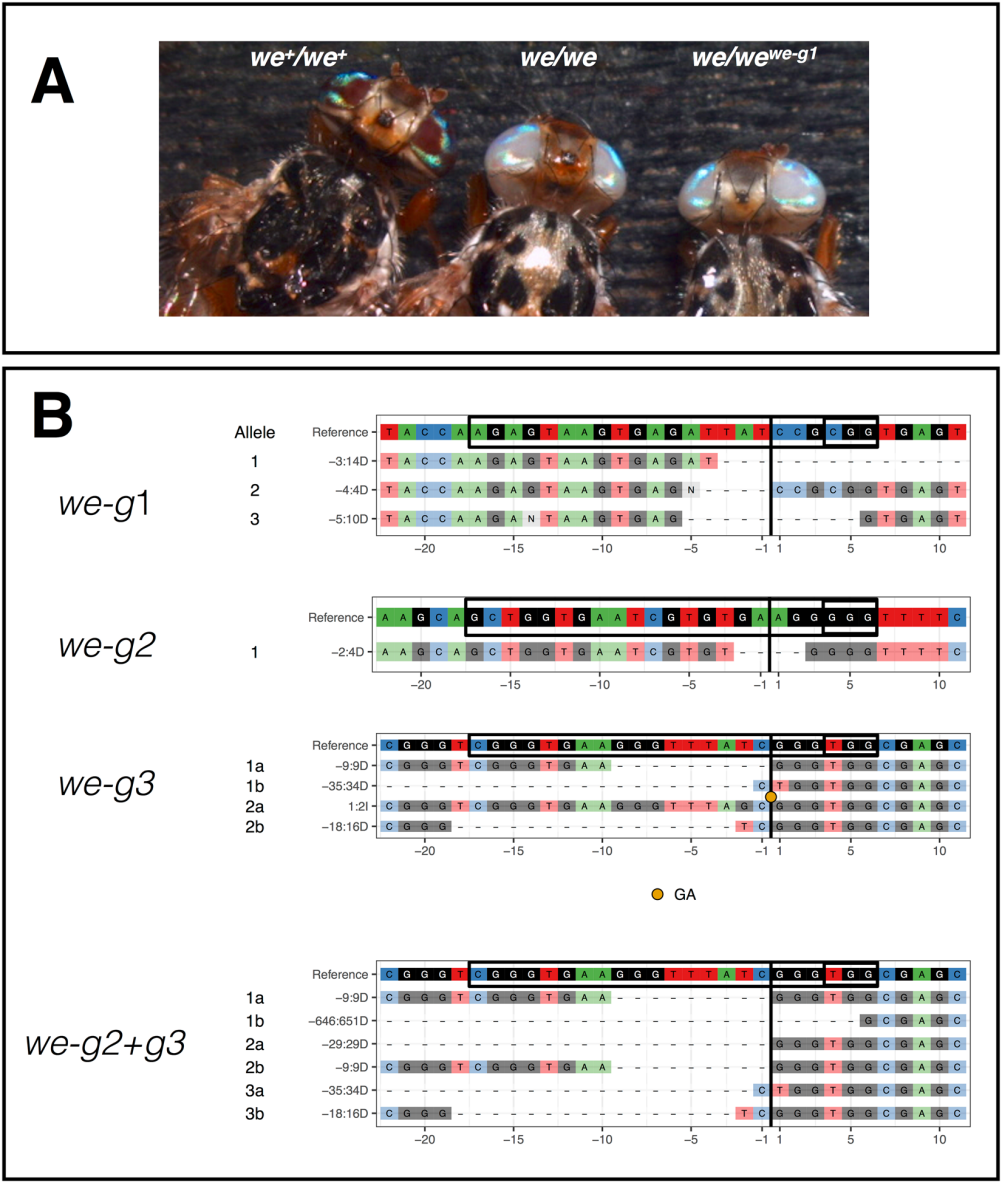
CRISPR induced *we* non-complementing alleles transmitted to the G1 progeny. (**A**) homozygous wildtype *we* ^+^ individual (left), homozygous mutant individual *we* (middle), G1 individual heterozygous for mutant *we* and CRISPR induced *we* mutation. (**B**) CrispRVariants plots of G1 mutant progeny of G0 targeted by *we-g*1 (3 sequences), targeted by *we-g*2 (1 sequence), *we-g3* (6 sequences) or *we*-g2 + g3 (4 sequences).

To test for germline transmission of Cas9-induced *we* alleles, 9 injected G0 red eyed flies (3 *we*-g1 RNP- and 6 *we*-g2 RNP-injected flies; Supplementary Table 3) were individually crossed with *we*-mutant partners (carrying *w1* alleles having a frameshift mutation in the sixth exon^41^; GenBank AH010565.2). Seven injected flies sired G1 progeny, six of which gave rise to mutant *white eye* flies **(**Fig. 2A **)**, with a highly variable transmission rate (1.5–100%, on average 52%; Supplementary Table 3). Non-complementation of the CRISPR-Cas9-induced mutations confirms that they are allelic to the original *we* mutation (Fig. 2A). Of the three *we*-g1 injected individuals, two males sired small batches of progeny in which 100% (*we-*g1#1; 6 flies) and 45% (*we-*g1#2*; 10 out of 22*), respectively, displayed the mutant phenotype (Supplementary Table 3). Of the six *we*-g2 injected individuals, 4 males produced various proportions of G1 mutant white-eyed progeny. Remarkably, the *we*-g1#1 and *we*-g2#5 lines gave rise to 100% and 98% G1 white-eyed offspring, respectively (Supplementary Table 3).

We randomly chose 4 out of 16 (6 + 10) G1 *we-*g1 mutant flies (two from *we-*g1#1 cross and two from *we*-g1#2 cross) and 2 out of 166 (53 + 113) *we-*g2 mutant G1 flies (one from *we-*g2#4 line and one from *we-*g2#5 line) (Supplementary Table 3). Sequencing of cloned PCR products revealed that the 4 *we-*g1 G1 flies inherited one allele from the *we* parent and three different *we* ^*CRISPR*^ alleles from the injected G0 fathers. We found an identical *we* deletion of 14 bp (labeled “−3:14D” in Figs 1E and 2B) in both analyzed flies from the *we-*g1#2 cross, suggesting that they inherited the same mutation from their common male founder (Fig. 2B: *we*-g1). Mutant G1 flies of the *we-*g1#2 line carried two different alleles (4 bp and 10 bp deletions; −4:4D and −5:10D, respectively). All three Cas9-induced alleles are small deletions, causing frame-shifts in exon 2 of the *we* coding region, and failed to complement the original *we* mutation. Two *we*-g2 targeted G1 mutant flies carried two novel alleles, one with a 4 bp deletion (*we*-g2#4 line; Supplementary Table 3) and one with a long 84 bp deletion (*we*-g2#5 line; Supplementary Table 3) (Fig. 2B: *we*-*g2*; 84 bp deletion not shown).

To study genes for which no mutant alleles are available, it may be useful to screen for mutant phenotypes by *inter se* crossings of G0 individuals in which the germ line has been targeted by CRISPR-Cas9. As a proof of principle, we injected *we*-g3 or *we*-g2/*we*-g3 RNPs and we obtained G0 individuals to be crossed. All the surviving G0 flies, 20 and 38 respectively, did not show somatic mosaicism in the eyes (Supplementary Table 2; 3^th^ and 5^th^ rows). When crossed *inter se*, we recovered two mutant individuals out of 26 G1 flies (8%) from the *we*-g3 G0 cross and three out of 184 flies (2%) from the *we*-g2/*we*-g3 G0 cross, demonstrating the feasibility of this strategy (Supplementary Table 4).

Sequencing of the targeted regions in 2 G1 flies of the *we-g3* cross identified, as expected, mutant *we* alleles. 2 out of 3 tested G1 flies of the *we-g2/we-g3* cross all exhibit identical 9 bp deletion *we* allele at *we*-g3, most likely derived from the same G0 injected parent (Fig. 2B: *we*-g3). One of these 3 flies carried a large deletion of 651 bp resulting from duplex targeting with *we*-g2/*we*-g3 RNPs (Fig. 2B).

### Cas9-induced somatic disruption of the *Ceratitis paired* gene

To test the functionality of purified Cas9 protein we established a fast *in vivo* assay by targeting the *Ceratitis* zygotic segmentation gene *Ccprd*, which is expected to give visible phenotypes in early larvae (within 3–4 days from the injections day).

**Figure 3:**
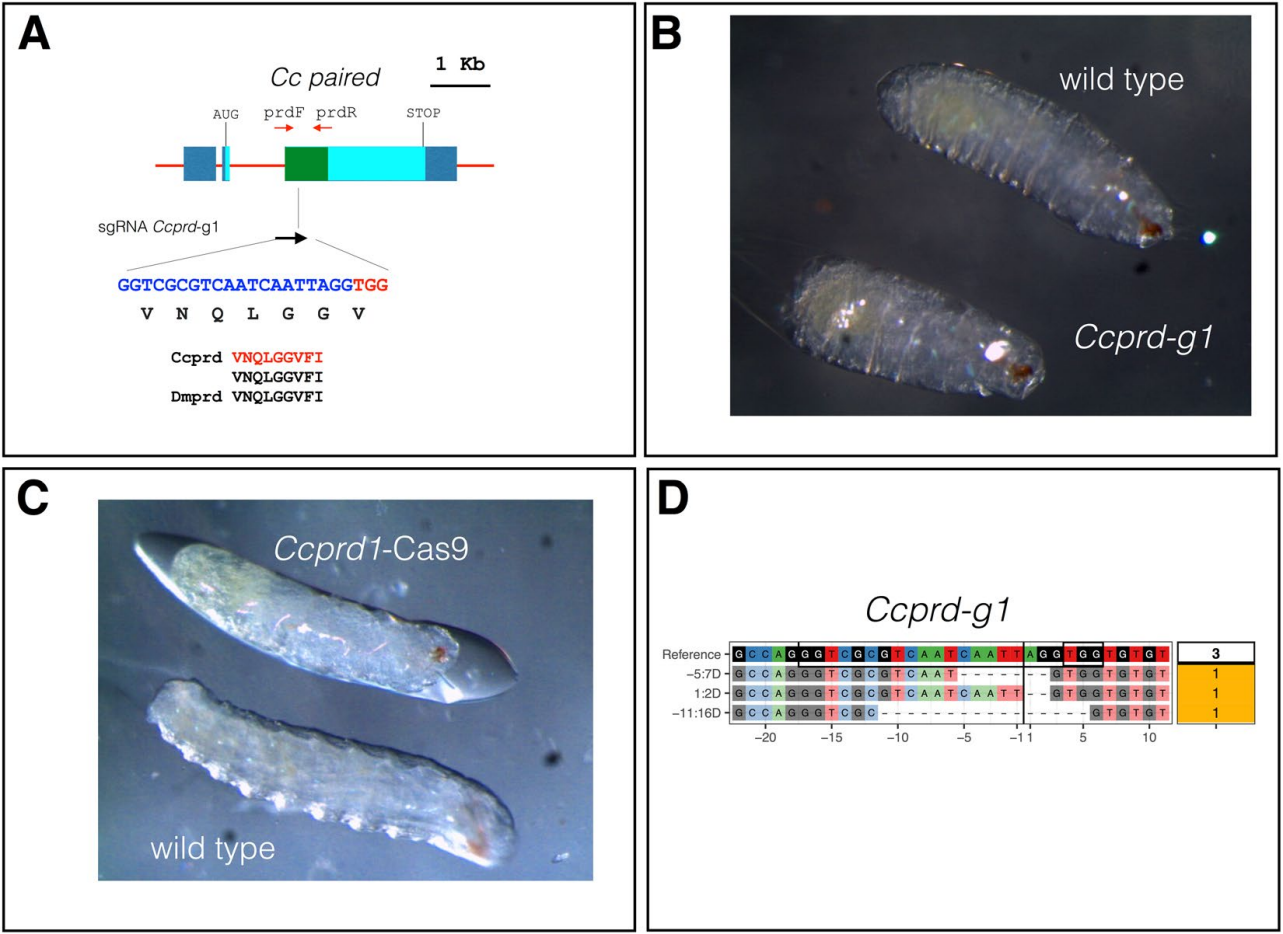
CRISPR-Cas9 targeting of *Ccprd*. (**A**) A scheme of the *Ccprd* gene and the positions and sequence of a sgRNA (**B**) Comparison of the cuticular morphology of a non-injected control (wild type) and a *Ccprd-g1* targeted embryo (*prd1*-Cas9). The injected embryo is significantly shorter and displays a twofold reduction in segment number reminiscent of the pair-rule phenotype described in *Drosophila* ^42, 43^. (**C**) After injection of *Ccprd*-g1, high variability in phenotypes was observed ranging from wildtype appearance to strong deformations and size reductions along the main body axis. (**D**) A CrispRVariants plot of *Ccprd* alleles found in larvae developed from embryos injected with RNP containing *Ccprd-g1* sgRNA.

We used CHOPCHOP^47^ to select one target sequence in *Ccprd* (*Ccprd*-g1) in a region encoding the conserved Paired domain (green box; Fig. 3A). Two injection rounds with *Ccprd-g1* (300 mM KCl) resulted in survival rates of hatching embryos of 35% and 24%, respectively (Supplementary Table 5). A slightly more pronounced lethality was caused by *Ccprd*-g1 (larval survival rate of 0.24–0.35; Supplementary Table 5) compared to the *we*-g1 and *we*-g2 RNPs effects (larval survival rates of 0.56 and 0.43, respectively; Supplementary Table 2). Comparing these values (0.24–0.35; Supplementary Table 5) to the larval survival rate of 0.46 with injection of buffer alone (Supplementary Table 5), RNP-mediated targeting of *Ccprd* led to a ∼0.1–0.2 higher mortality. Approximately 1–5% of the injected embryos showed either delayed development (late hatching rate) or arrest at late stages of embryogenesis, while none of these effects were observed upon injection of Cas9 alone (data not shown). Some lethality was observed at larval stages, with few individuals showing impaired locomotor activity and abnormal cuticular morphology. Only 5–11% of injected individuals developed to adulthood, with a delay of 1–2 days compared to flies injected with buffer alone or with *white* targeting RNPs (Supplementary Table 5).

In these 2 sets of injections, 90 embryos and 160 embryos failed to hatch into larvae, which is approximately 90% (Supplementary Table 5) and apparently displayed disorganized cuticular/internal structures. Some were up to 50% shorter in size compared to control embryos and consisted of a reduced number of segments (Fig. 3B). Some developed into mutant larvae, which, although able to move, failed to hatch (Fig. 3C). Sequencing of individuals injected with *Ccprd*-g1 unveiled lesions in the targeted regions of the *Ccprd* locus (Fig. 3D). We conclude that *Ccprd-g1* targeting was effective in inducing lesions in *Ccprd* and causing embryonic developmental malformations.

## Discussion

Over the last two decades, novel genetic strategies in pest insect management have been developed to improve their effectiveness in the field. Genetic technologies used thus far in the medfly are based on random integration of transposable elements into the genome^4^, site-specific modification of the randomly integrated transgene^10^ and embryonic or transgene-mediated RNA interference (RNAi)^13, 15^. The disadvantage is that such genetically modified medflies must be continuously tested with respect to fitness and competitiveness as well as to stability and expression of the transgene^9, 10^. The CRISPR-Cas9 technology offers a novel approach to stably introduce exogenous DNA sequences at preselected locations in the genome. The use of preloaded Cas9-sgRNA complexes has already been successfully applied to target genes for disruption in a growing number of insect species, including 5 dipteran^28, 33, 34^ and 4 lepidopteran^35–38^ species.

To our knowledge, this is the first report showing that the Cas9-sgRNA RNP complexes can be used to effectively mutate genes in a major agricultural dipteran species such as *Ceratitis capitata*. We report here that Cas9-mediated NHEJ events generate lesions in the *we* and *Ccprd* genes. Targeting the *we* gene caused red-white eye mosaicism up to 50% of injected individuals, indicating a high rate of somatic bi-allelic loss of function mutations. Strong variability of somatic mosaicism was observed in the 5 injections experiments, using either single sgRNAs (*we*-1, *we*-2 and *we*-3) or in combinations (*we*-1/*we*-2 and *we*-2/*we*-3). This could be due to different efficiencies in target recognition of the sgRNAs, (see Supplementary Table 2). Other studies have reported success rates varying between 10% and 70% on a sgRNA-to-sgRNA and gene-to-gene basis^30^.

We demonstrated that Cas9 RNPs with *we*-1 or *we*-2, can very effectively mutate primordial germ cells with transmission rates up to 98–100% in 2 out of 7 crosses. To our knowledge, this germline transmission rate is the highest achieved in insects with purified Cas9, either commercial or lab-produced succeeding those observed in *Drosophila* (17%)^28^, *Aedes aegypti* ^30, 33^ (in these 2 studies, respectively 21% and 90%), *Anopheles funestus* ^34^ (67%), and *Spodoptera littoralis* ^37^ (43%).

These differences in efficiency can be explained by various reasons. For example, proficiency in injecting embryos manually can vary greatly. Consistent delivery of a given amount of RNPs is another serious limitation of this technique, since manual injection of the components into the insect embryos, is technically demanding and has an inherently low throughput. For our injections, we used a microinjector with oil pressure to ensure a constant flow of the RNP mix. Lab-pulled needles, of which the tips were manually broken, generate variability in how much volume reaches in the embryos. The use of microinjectors with controlled pressure and standardized needles can help to improve reproducibility and reliability. Another important issue is stability and solubility of Cas9 protein, as previously observed in zebrafish^39^. We report here that adding KCl at 300 mM to the injection mix may also increase Cas9 efficiency in *Ceratitis*; further experiments against more targets will be needed to corroborate this conclusion.

As rearing insect species, like *Ceratitis*, is often more laborious than the model *D. melanogaster*, the possibility to screen for mutant individuals bearing 2 loss of functions alleles in G_1_ by crossing RNP injected individuals, is a very attractive solution. Our data show that we can obtain 2–8% of mutant *we* G1 flies, by *inter se* crossings of G0 RNP-injected individuals in less than 2 months.

Several studies have reported that simultaneous use of two Cas9-sgRNAs is an effective means to generate deletions between the two targeted sites^24, 29, 39, 48^. While *we*-g1 and *we*-g2 RNPs were individually effective in gene editing, the absence of a deletion spanning the two targeted sites less than 100 bp apart in 4 different series of experiments (at 150 mM and 300 mM KCl) may be due to reciprocal steric hindrance that prevents that two adjacent Cas9-sgRNA complexes can cut both sites simultaneously. One study has shown that two loci separated by 119 bp could be cleaved simultaneously in cultured human cells but only at a low efficiency^49^. In contrast, RNPs targeting simultaneously two sites more distant from each other, such as *we*-g2 and *we*-g3 (489 bp), were effective in generating long deletions in both somatic and germ-line cells. Hence, it is conceivable that a multiplex CRISPR-Cas9 system can be used to remove, for instance, protein domains encoded by single exons in the medfly as long the targeted sites are more than 100 bp apart.

Furthermore, to test the activity of different Cas9 protein batches we established an *in vivo* assay based on targeting the *Ceratitis* zygotic segmentation gene *Ccprd*. This test produces detectable phenotypes within 3 days after injection and presence of NHEJ lesions can be validated by subsequent sequence analysis. The segmental defects observed in the affected *Ceratitis* embryos are reminiscent of the *pair-rule* phenotype of *Drosophila* ^42, 43^. The phenotypic variability is likely a result of variable proportions of wildtype cells (*Ccprd* ^+^ */Ccprd* ^+^ or *Ccprd* ^+^ */Ccprd* ^*−*^) and mutant cells (*Ccprd* ^*−*^ */Ccprd* ^*−*^) in these affected individuals. The distinct shortening in length and reduction of segments in some individuals suggest that biallelic targeting was very effective and produced large mutant clones.

Cas9-sgRNA complexes seem to be rapidly degraded in *Drosophila* ^28^, often within hours after administration; Cas9 protein is degraded within 24 h after being applied to cultured human cell lines^17^. As the short-lived activity of Cas9 prevents the induction of late mutational events, this may help to reduce off-target effects^37, 39, 50^. On the other side, its high efficiency may lead to biallelic mutations in most, if not all, dividing embryonic cells.

Our DNA-free method, which does not depend on delivery of Cas9 by plasmid or transgene, offers a possible solution to bypass existing regulatory restrictions that prevent the release of genetically modified organisms in the field. Genome modifications with the use of purified Cas9 endonuclease may be more acceptable to the public and considered legally legitimate by governments. As an example, with this approach novel sexing strains can be developed to produce male-only progeny in *Ceratitis* and other Tephritidae for pest control programs based on the sterile insect technique. This technique relies on the introduction of excessive numbers of only males in infested areas and hence requires large-scale sorting of males and females prior to release. In *Ceratitis capitata* and various *Bactrocera* species, this is achieved with the use of dominant phenotypic markers which are linked to the male determining Y chromosome. CRISPR-Cas9 provides an effective means to introduce new markers on the Y that may improve existing selections methods or even establish selection methods in species where no such markers were hitherto available^51^. Another potential application to improve of SIT is to develop a system that transforms genotypical females into phenotypical males. For instance, in *Drosophila melanogaster* a temperature-sensitive (*ts*) mutation (a single amino acid substitution) in the sex determining gene *transformer-2* exists that causes XX individuals to develop into males at the restrictive temperature^52^. This gene is structurally and functionally conserved in a wide range of insects. Transient embryonic RNAi of *tra-2* in several Tephritidae species, including medfly^8^, the Mexican fly *Anastrepha suspensa* ^53^ and the oriental fly *Bactrocera dorsalis* ^54^ led to complete sexual transformation of XX individuals into phenotypically normal males. Hence, it may be feasible to introduce the identical mutation in *tra-2* orthologs to generate *ts* mutants that produce male-only progeny following heat shocks during development. Taken together, this novel and highly effective editing technology can be applied to produce new and more effective strains in pest control management and, given that this technology is free of exogenous DNA, it may in the long term foster global acceptance regarding the release of genetically modified pest insects^31, 55–57^.

Finally, we would like to make a point that the gene targeting protocol presented in this study is simpler and more feasible than those published in other studies for the following reasons: (1) lab-produced Cas9 protein is significantly cheaper than commercially available products; (2) pre-assembled Cas9 and gRNAs complexes are likely to be more stable in embryos than, for instance, Cas9 mRNA; (3) Cas9 mRNA production is time consuming and expensive, and can be stored only for few weeks, while purified Cas9 protein can be stored at −80 °C for years, and pre-assembled RNPs can be stored at -80 °C for at least 10 weeks without losing their activity; (4) finally, pre-assembled RNP complexes may have less off target effects^39, 50^. Our protocol and the purified Cas9 protein have been already successfully applied in the housefly, *Musca domestica*, to target the *Drosophila yellow* ortholog *brown body* ^58^ and the male determining gene *Mdmd* ^59^.

Our RNP-based procedure will be helpful in investigating natural traits of this major agricultural pest, for example invasiveness and host adaptation, reproduction, olfaction (fruit seeking behavior), chemoreception, toxin and insecticide metabolism^60^. The recent availability of the medfly genome sequence^60^ combined with the successful implementation of CRISPR genome editing technology, as reported here, opens the road to transfer basic knowledge to applied research. Next challenges for the CRISPR-Cas9 technology in the medfly will be to exploit homology-directed recombination for genome editing, either to insert transgenes in specific regions or to modify DNA sequences according to a co-injected template. The ability of Cas9 to perform efficient recombination at specific genomic positions will greatly advance progress towards novel genetic strategies for control of pest insects, such as gene drive which is based on integration of self-propagating cassettes^61–64, 65^.

## Methods

### Rearing of *Ceratitis capitata*

The medfly Benakeion and *we* strains were reared in standard laboratory conditions at 25 °C, 70% relative humidity and 12 h/12 h light–dark cycles. Adult flies were fed with a mixture of yeast and sucrose powder (1 v/2 v). Eggs were collected in dishes filled with water and after hatching transferred to larval food. Pupae were collected and stored in dishes until eclosion.

Following strains were used in this study: (a) the wildtype strain Benakeion which was obtained from P. A. Mourikis (Benakeion Institute of Phytopathology, Athens, Greece) and (b) the mutant *we/we* strain Benakeion^4^ kindly provided by Kostas Bourtzis (Pest Control of FAO/IAEA, Seibersdorf, Austria).

### Medfly larval food

40 gr cellulose (couch paper roll), 30 g, sugar, 30 g, yeast extract, Cholesterol stock: 10 ml, HCl stock: 2 ml, Benzoic stock: 8,5 ml, water 400 ml.

Cholesterol stock: Cholesterol 5 g (BioChemica, AppliChem), distilled water 140 mL, ethanol 96% 50 mL (Carlo Erba).

HCl stock: distilled water 384 mL, HCl (37%) 66 mL (Carlo Erba). Benzoic acid stock: Benzoic acid 50 g (Carlo Erba), Ethanol 96% 300 mL, distilled water 150 mL.

### Cas9 protein purification

We produced our own supply of Cas9 endonuclease by expressing HIS tagged protein in bacteria^27^ and following the purification protocol described in Truppo *et al*.^45^ and Dathan *et al*.^46^. The *pET* plasmid which carries the recombinant His-tagged Cas9 gene was transformed into BL21(DE3) bacterial strain. Protein expression was induced in presence of 0.5 mM isopropyl-β-D-thiogalactopyranoside (IPTG) for 16 h at 22 °C. 100 ml cell pellet was resuspended in 10 ml of cold lysis buffer (50 mM Phosphate, 500 mM NaCl, 10 mM Imidazole, 1 mM DTT, pH 8) supplemented with protease inhibitor mixture (1 mM PMSF and 1.0 mg/ml of lysozyme) and incubated at room temperature for 30 min. Cells were disrupted by sonication on ice with 10 s on and 10 s off cycles for 10 min. After centrifugation at 14,000 rpm for 30 min at 4 °C, supernatant was purified by FPLC (AKTA) on a 1 mL His Trap HP column. The column was washed with lysis buffer and bound protein was eluted using a gradient from 10 mM to 500 mM imidazole. Protein elution was monitored by measuring absorbance at 280 nm and resulting fractions were analyzed by 15% SDS-PAGE. The eluted fractions were dialyzed against buffer (20 mM HEPES, 150 mM KCl, 1 mM DTT, 10% glycerol, pH 7.5) and further purified and analyzed on a Superdex 200 increase 10/300 GL column.

### sgRNA synthesis and RNP complex assembly

sgRNA were designed using CHOPCHOP^47^ https://chopchop.rc.fas.harvard.edu/. CHOPCHOP lists the Target Sequence (including the PAM), the genomic location of the target, the strand (- or +), the GC content of the guide and the Off-targets. Templates for sgRNA production were designed as previously described^25^.

Oligos were obtained from Life Technologies and Sigma, as standard primers except where otherwise noted. sgRNA DNA templates were oligo-based and obtained by PCR as previously described^25^ (), using the sgRNA forward primer (see below) for T7 templates GAAATTAATACGACTCACTATA-N19/20-GTTTTAGAGCTAGAAATAGC (with N19/20 indicating the target site either 19 or 20 nt long) and the invariant reverse primer AAAAGCACCGACTCGGTGCCACTTTTTCAAGTTGATAACGGACTAGCCTTATTTTAACTTGCTATTTCTAGCTCTAAAAC (PAGE-purified, Life Technologies).

Forward primer sequences (Cas9 target sequence is underlined):

*we*-g1:

GAAATTAATACGACTCACTATAGAGTAAGTGAGATTATCCGGTTTTAGAGCTAGAAATAGC

*we*-g2:

GAAATTAATACGACTCACTATAGCTGGTGAATCGTGTGAAGGGTTTTAGAGCTAGAAATAGC

*we*-g3: GAAATTAATACGACTCACTATACGGGTGAAGGGTTTATCGGGGTTTTAGAGCTAGAAATAGCC

*cprd*-g1: GAAATTAATACGACTCACTATAGGTCGCGTCAATCAATTAGGGTTTTAGAGCTAGAAATAGC

Primer extension was performed using Q5® High-Fidelity DNA Polymerase (NEB). The DNA template was cleaned by phenol/chloroform and chloroform extractions and precipitated in ethanol 70% at −20 °C overnight. Following centrifugation at 14,000 rpm for 15 min at 4 °C, the pellet was resuspended in 40 μl ddH_2_O. For synthesis of sgRNA we followed the instructions of Megatranscript T7 kit (Ambion) using 400 ng of target template and the reaction run at 37 °C overnight, followed by ammonium acetate precipitation as per the manufacturer’s protocol and as described previously^25, 39, 50^. After RNA synthesis, DNA template was removed by incubating with TurboDNase (Mmessage mMACHINE T7 Ultra Kit, Ambion) for 15 min at 37 °C. Before use, all sgRNAs were quality-controlled on denaturing 2.5%MOPS gels.

The reaction for complex formation was prepared by mixing 1.8 µg of purified Cas9 protein with approximately 1 µg of sgRNA in a 5 μl volume containing 150 or 300 mM KCl, according to the protocols proposed by Lee *et al*.^28^, and Burger *et al*.^39^, respectively. The freshly prepared mixture was incubated for 10 min at 37 °C for pre-assembly of RNP and kept on ice prior to injections.

### Microinjection of sgRNA-Cas9 RNP complexes

Embryos of the wildtype Benakeion strain were collected 1 hour after egg laying and chorion membrane was manually removed. Dechorionated embryos were aligned and glued to 3MM double side tape on a cover slip with posterior ends pointing to injection site and covered with Oil S700 (Sigma). The first injection of 240 embryos with *we*-g1 RNA was performed in the Bopp lab in Zurich University (Switzerland), using an electronic microinjector FemtoJet Eppendorf and related commercial needles ready to use. The remaining embryos injections were performed in Saccone lab (Naples University), with the following equipment and procedures: glass needles were pulled with a Narishige PB-7 and manually broken to produce sharp edges at the injection tip. The needle was filled with 1 μl of preloaded sgRNA-Cas9 mix and injected into the posterior end of embryos. A Narishige IM-9B oil microinjector was used to produce a constant flux of the aqueous mix.

A Leica inverted microscope DM-IRB was used for micro-injections. After injection, excess of oil was carefully removed and cover slips were put on an agar plate overnight at 25 °C. Surviving larvae were transferred after 24 hours to petri dishes containing medfly larval food. A Jove video is available, describing a similar method of medfly embryos microinjection^49^.

Phenotypic analyses of mutant individuals were conducted using Leica MZ16F stereomicroscope. For the *we* targeting, we screened for red-white eye mosaicism in G0 adult flies and for white eye phenotype in G1 flies; for the *Ccprd* targeting we screened for embryonic/larval mutants in the injected G0 individuals.

### Genomic analysis of Cas9-mediated DNA lesions

DNA extraction was performed, with minor modifications, according to the protocol of Holmes and Bonner^66^. Individual larvae or adult flies were placed in a 1.5 ml Eppendorf tube and manually crushed with a pestle in 200 μl Holmes Bonner buffer (urea 7 M, Tris-HCl 100 mM pH 8.0, EDTA 10 mM pH 8.0, NaCl 350 mM, SDS 2%). Subsequently, DNA was purified by phenol/chloroform extraction, followed by chloroform extraction and ethanol precipitation. The pellet was resuspended in 30 μl or 100 µl water containing RNaseA. The resulting DNA was used as template to amplify the region encompassing the target sites, using the following primers:

F-*we*: GCCCTACGAGCAATCCTCT

R1-*we*: TCTGCAATGAGCGTCATATAC

R2-*we*: TTCTGCGATAGCTTTTTCAACA

F-*Ccprd*: CTTCGACACACAACCGTGTG

R-*Ccprd*: AGAATGCTTGTGGGAATGTTCT

DreamTaq (Life Technologies) polymerase was used for PCR amplifications according to the manufacturer’s instructions. The PCR products were purified with StrataPrep PCR Purification Kit (Agilent Technologies) and subcloned using StrataClone PCR cloning Kit (Agilent Technologies). Positive clones were sequenced by Sanger method and ABI 310 Automated Sequencer (Applied Biosystems). CrispRVariants and panel plots were compiled from primary Sanger sequencing data as described in Lindsay *et al*.^67^as well as using new functionality to display duplex guide pairs (e.g., Fig. 1F) as implemented in the development version 1.3.6.

### Data availability

All data generated or analyzed during this study are included in this published article (and its Supplementary Information files).

## Electronic supplementary material

Supplementary Tables 1-5 and Suppl. Fig 1
