# Supplementary material for: Highly efficient DNA-free gene disruption in the agricultural pest Ceratitis capitata by CRISPR-Cas9 ribonucleoprotein complexes
Source: Sci Rep. 2017 Aug 30;7:10061. doi: 10.1038/s41598-017-10347-5 (PMC5577161; doi:10.1038/s41598-017-10347-5)
Supplement: Supplementary file 1 — Supplementary Tables 1-5 and Suppl. Fig 1 [file 41598_2017_10347_MOESM1_ESM.pdf]

Supplementary Materials for

Highly efficient DNA-free gene disruption in the agricultural pest *Ceratitis capitata* by CRISPR-Cas9 ribonucleoprotein complexes

Angela Meccariello[1], Simona Maria Monti[2], Alessandra Romanelli[3], Rita Colonna[1], Pasquale Primo[1], Maria Grazia Inghilterra[1], Giuseppe Del Corsano[1], Antonio Ramaglia[4], Giovanni Iazzetti[1], Antonia Chiarore[5], Francesco Patti[5], Svenia D. Heinze[6], Marco Salvemini[1], Helen Lindsay[6,7], Elena Chiavacci[6], Alexa Burger[6], Mark D. Robinson[6,7], Christian Mosimann[6], Daniel Bopp[6] and Giuseppe Saccone[1,*]

*correspondence to: giuseppe.saccone@unina.it
Supplementary Tables 1- 5
Supplementary Fig. 1

Cas9 µg/µl	Injected embryos	Larvae/ Embryos	Pupae/ Larvae	Adults/ Pupae	Adults/ Embryos
0	114	0.56(64)	0.84(54)	0.66(36)	0.31(36)
0.9	150	0.59(88)	0.37(33)	0.82(27)	0.18(27)
1.8	110	0.57(63)	0.49(31)	0.68(31)	0.28(31)
3.6	134	0.51(69)	0.41(28)	0.71(20)	0.15(20)

Supplementary Table 1: **Survival rates after injecting recombinant Cas9 protein into embryos.**
Numbers represent the percentage of survivors (actual number in parentheses) for a given specific developmental stage relative to number of individuals of the preceding stage. The last column is the adult survival rate with the respect of the injected embryos.

Cas9 µg/µl	sgRNA 0.2 µg/µl	Injected embryos	Larvae/ Embryos	Pupae/ Larvae	Adults/ Pupae	Adults/ Embryos	Somatic mosaics
1.8	*we*-g1	240	0.56(134)	0.33(34)	0.18(6)	0.02(6)	0.5(3)
1.8	*we*-g2	180	0.44(80)	0.36(29)	0.83(24)	0.13(24)	0.04(1)
1.8	*we*-g3	200	0.46(92)	0.34(31)	0.64(20)	0.1(20)	0
1.8	*we*-g1/ *we*-g2	240	0.43(104)	0.44(44)	0.95(42)	0.17(42)	0.23(10)
1.8	*we*-g2/ *we*-g3	250	0.49(123)	0.53(58)	0.65(38)	0.15(38)	0

Supplementary Table 2: Survival rates and frequencies of individuals with somatic mutant clones after injecting *we*-g RNPs.

Numbers represent the percentage of survivors (actual number in parentheses) for a given specific developmental stage relative to number of individuals of the preceding stage (except 7th column, which shows the adult survival rate with the respect of the injected embryos). 31 larvae which survived *we*-g1 injections (row 1), 5 larvae of *we*-g1/*we*-g2 (row 4) and 13 larvae of *we-g2/we-g3* injections (row 5) were sacrificed for molecular analysis.

Line	Sex of founder	*we/we*^{CRISPR} flies	*we/we*$^{+}$ flies	Transmission rate
we-g1#1	M*	6	0	**1**
we-g1#2	M*	10	12	0.45
we-g2#3	F	0	55	0
we-g2#4	M	53	34	0.6
we-g2#5	M	113	2	**0.98**
we-g2#6	M	1	68	0.01
we-g2#7	M*	14	10	0.58

Supplementary Table 3: Transmission of CRISPR-induced *we* mutations in the germ line.
From 9 injected G0 flies 7 (RNPs *we*-g1 or *we*-g2; see first column) produced progeny with *we* phenotype when crossed to *we/we* mutant partners (*we*= mutant white eye, *we*^{CRISPR}= novel mutant *we* alleles, *we*$^{+}$ = wild type allele). 2 flies were sterile. The highest transmission rates of CRISPr induced mutant *we* alleles (100% and 98%) are shown in bold. The asterisks indicate G0 founders showing eye mosaicism.

sgRNAs	G0 injected parents	*we*^{CRISPR} flies	*we*$^{+}$ flies	Total adult G1 flies	Mutants frequency
we-g3	8M x 12F	2	24	26	0.08
we-g2+ *we*-g3	18M x 20F	3	181	184	0.02

Supplementary Table 4: Germline transmission of CRISPR-induced *we* mutations.

Progeny of two families of G0 injected adults are shown. All G0 injected individuals from each of the 2 injection experiments shown in Table 2, were crossed and G1 scored for *we* mutant flies.

Cas9 µg/µl	sgRNA µg/µl	Injected embryos	Larvae/ Embyos	Pupae/ Larvae	Adults/ pupae	Adults/ Embryos
0	0	172	0.46(80)	0.74(59)	0.97(57)	0.33(57)
1.8	0	110	0.57(63)	0.84(54)	0.68(31)	0.28(31)
1.8	prd1 0.2	164	0.35(58)	0.17(10)	0.8(8)	0.05(8)
1.8	prd1 0.2	244	0.24(58)	0.5(29)	0.96(28)	0.11(28)

Supplementary Table 5: Survival rates after injecting of *Ccprd*-g1 RNP into syncytial embryos.

First two rows show the survival rates in the controls with buffer only and with Cas9 only. Numbers represent the percentage of survivors (actual number in parentheses) for a given specific developmental stage relative to number of individuals of the preceeding stage. The last column is the adult survival rate with the respect of the injected embryos.

LOCUS GAMC01015788 mRNA

CAGCAGCAGCAGCAATGGCAATATGCCTCTTCACCTATTGTTCAACACGCAAACGGACGGTGCTCAGTTT
GTTGCAGTCAATGGTTGTACATAGTACCACTCCGGTTTAGTGCGTAATAATTAGTGCTGTCTTAGACTGA
TAAACAAGAAATAAAAAAAATAGAGAAAAATTGTTGAAGCAGAGCAAATTACGACTCTTTGCCAGCAGAC
AGCAAGCAAAAAACAAGCAATTACAAAAAAAAATACCAAAAAACCGAACGAGAAAAATAAAAAATAAAAA
TTAAGAAAAAAATTTTGCAATAAAAAAGTGAAAAAACTGCTAAAAAAACTGTTAAAAATTTACAAAAAAA
AAACATAAAATAACCTAAAAGTAGAGACACAGGATAGTAGATACTAAAGCAGTAGAACGCCATAGAGCAA
AAAAAAAATTCAAATGGGTCAGGAGGATCAGGAGGTGTTAATAAGAGGCGGCAAGGCCACTAGCACATCG
GCCGAGAGTTTGAATAACAACAACGAGCAGCCCTACGAGCAATCCTCTATAAATCAGGGATTTAGCAAAA
ATTATGGCACACTCTCACCGCCATCGCCTGCACTCA**CCGCGGATAATCTCACTTACTC**TTGGTACAATTT
GGACGTCTTCGGCGCTGTACATCAGCCCGGCTCGAGTTGGAAGCA**GCTGGTGAATCGTGTGAAGG**GGGTT
TTCTGCAATGAGCGTCATATACCCGCGCCGCGCAAACATCTCTTGAAAAACGTCTCTGGCGTCGCTTACC
CCGGTGAATTACTTGCTGTAATGGGCAGTTCTGGTGCAGGCAAAACTACACTTCTCAATGCGATTGCCTT
TCGTTCATCGAAGGGCGTGCAAATTTCGCCCTCCACCATTCGCATGCTCAACGGTCATCCCGTTGATGCA
AAGGAAATGCAGGCACGCTGTGCTTATGTTCAGCAGGATGATCTTTTCATTGGTTCGCTCACCGCACGTG
AGCATCTTATCTTCCAGGCAATGGTGCGCATGCCACGGCATATGACACAAAAACAGAAAGTACAACGTGT
CGATCAAGTTATACAGGACCTCTCGCTGGGTAAATGTCAGAATACGTTGATTGGCGTGCCGGGT**CGGGTG**
AAGGGTTTATCGGGTGGCGAGCGTAAGCGACTGGCATTCGCCTCGGAGGCGTTAACCGATCCGCCGCTAT
TAATTTGCGATGAACCTACCTCGGGTTTGGACTCGTTCATGGCACACAGCGTCGTACAGGTGTTGAAAAA
GCTATCGCAGAAAGGCAAAACTGTTATATTGACCATACATCAACCATCCTCGGAGTTATTTGAACTTTTC
GACAAAATATTACTTATGGCAGAGGGTAGAGTTGCCTTTCTGGGTACACCCGGTGAAGCGGTGGACTTTT
TCTCATACATTGGCGCTACTTGTCCAACCAATTACAACCCAGCAGATTTTTACGTACAAGTTTTGGCTGT
AGTACCAGGTCGGGAAGTGGAATCACGTGAACGTATAGCCAAGATATGTGATAATTTTGCAGTCGGAAAG
GTCTCTCGCGAGATGGAGCAAAATTTTCAAAAGCTGGTTAAGTCCAATGGCTTTGGTAAGGAGGACGAAA
ACGGATACACATACAAAGCTTCCTGGTTCATGCAATTCCGTGCCGTACTTTGGCGTTCTTGGCTGTCGGT
ATTGAAAGAACCGTTATTAGTTAAAGTGCGCCTATTACAAACGACGATGGTTGCTGTTCTCATTGGACTA
ATCTTTCTGGGACAACAATTAACCCAAGTCGGTGTTATGAACATTAACGGCGCCATTTTCTTGTTTCTGA
CCAACATGACTTTCCAAAATTCCTTCGCTACCATAACTGTCTTCACCACCGAACTGCCTGTCTTCATGCG
TGAGACACGTAGTCGTCTGTATCGTTGCGACACTTACTTCTTGGGTAAAACCATTGCCGAACTGCCGCTC
TTCTTGGTGGTACCGTTTCTCTTCACCGCCATCGCATATCCTCTCATTGGCTTACGTCCCGGTGTCGATC
ATTTCTTCACCGCTCTGGCGCTTGTCACCTTAGTAGCCAATGTTTCAACTTCCTTTGGTTATTTGATTTC
TTGCGCTTGCTCGTCGACATCAATGGCTCTATCGGTGGGTCCACCTGTTATTATACCATTTTTACTTTTT
GGCGGTTTCTTTTTAAACTCCGGCTCGGTGCCGGCGTACTTTAAATGGTTGTCGTACTTATCATGGTTCC
GTTATGCCAATGAAGGTTTACTGATTAATCAATGGGCAGATGTGAAGCCTGGAGAAATCACTTGCACTCT
ATCCAATACCACTTGCCCAAGCTCCGGTGAGGTTATATTGGAGACTTTGAATTTCTCAGCGAGTGATCTA
CCATTCGATTTCATTGGATTGGCGTTGCTGATTGTGGGCTTCCGAATATCCGCTTATATAGCGCTAACTA
TGCGCGCACGTCGAAAGGAATGAGTGGGGAAGGAGGAATGTCTAGACTATAAGATTTTTTGGTTTTAAAT
AATTAGTTTTGTACGAACACGCATTTATCATATAATAAATCGATAACTTTGGCAAGTTTATGTTTTATTT
ACTCTAAATGGCATTTGCAATGGAGCTGTAAACAAAA

Supplementary Fig. 1. Partial cDNA sequence of *we*. Positions of *we*-g1, *we*-g2 and *we*-g-sgRNAs are shown in bold with the PAM in red.
